# Bilateral pulmonary interstitial emphysema in a preterm infant on continuous positive airway pressure: clinical and radiological correlation

**DOI:** 10.1590/0100-3984.2016.0198

**Published:** 2018

**Authors:** Mariana Chiaradia Dominguez, Camila da Silva Pires, Mônica Carvalho Sanchez Stopiglia, Maria Aparecida Marques dos Santos Mezzacappa, Beatriz Regina Alvares

**Affiliations:** 1 Centro de Atenção Integral à Saúde da Mulher / Universidade Estadual de Campinas (Caism/Unicamp), Campinas, SP, Brazil; 2 Faculdade de Ciências Médicas da Universidade Estadual de Campinas (FCM-Unicamp), Campinas, SP, Brazil

**Figure 1:**
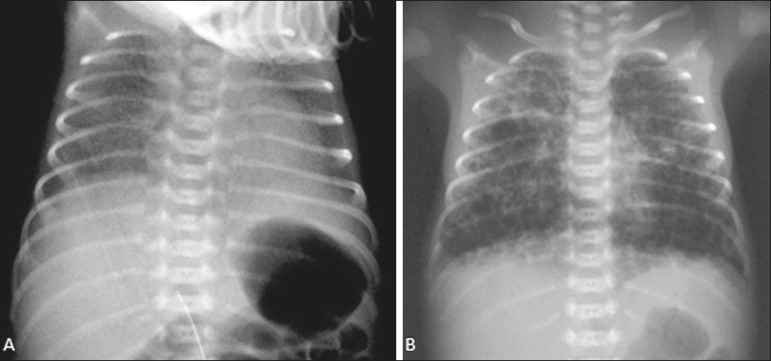
**A:** Lungs presenting bilateral diffuse reticulogranular infiltrate and peripheral air bronchograms, characteristic of RDS. **B:** Hyperinflation of the lungs, with cystic images of varying dimensions distributed bilaterally, consistent with PIE.

Dear Editor,

Here, we report the case of a newborn male, the product of a diamniotic twin pregnancy and a vaginal birth at 27 weeks and 2 days of gestational age, who had a birth weight of 920 g, with 1-, 5-, and 10-min Apgar scores of 3, 7, and 9, respectively. The mother did not receive corticosteroids, and there were no signs of infection in the amniotic fluid at the time of delivery. At birth, the neonate presented bradycardia and received mask ventilation with a positive pressure of 20 cmH2O. After 50 s, the heart rate returned to baseline, the infant then being transferred to the neonatal intensive care unit (NICU). At NICU admission, the infant was placed on continuous positive airway pressure (CPAP) at 5 cmH2O with a fraction of inspired oxygen of 50%. On physical examination, lung auscultation revealed normal breath sounds and few rhonchi. The neonate also showed moderate intercostal retractions and respiratory rate of 60 breaths/min. As can be seen in Figure 1A, the chest X-ray findings were consistent with respiratory distress syndrome (RDS). After five hours of life, lung auscultation showed a decrease in air intake and there were moderate intercostal retractions. At eight hours of life, he evolved to apnea, respiratory difficulty, grunting, and subdiaphragmatic retractions. A new chest X-ray showed diffuse, bilateral cystic images consistent with pulmonary interstitial emphysema (PIE), as depicted in Figure 1B. Endotracheal intubation was performed, the neonate subsequently evolving to bradycardia and cyanosis of the extremities. Adrenaline and calcium gluconate were administered. Cardiopulmonary resuscitation was performed, without success, for 20 min, and the infant died at nine hours of life.

PIE consists in the passage of air to the lung interstitium, radiologically characterized by tubular, tortuous radiolucent images, 2-3 mm in width, radiating from the hila to the lung periphery, as in the present case. PIE should not be confused with the peripheral air bronchograms seen in neonatal RDS, in which the bronchial branches show smaller diameters and a more regular configuration. The evolution can be rapid, cystic images forming within a few hours. In patients with PIE, tomography of the chest can show a pattern of lines and points in the middle of air collections, in addition to regular cystic formations, or pseudocysts^(1,2)^.

Risk factors for PIE include prematurity, the use of invasive mechanical ventilation and severe RDS^(3-5)^. To reduce the deleterious effects of invasive mechanical ventilation, nasal CPAP has been used with increasing frequency^(6)^. Nevertheless, data in the literature suggest that there are mechanisms related to the occurrence of PIE in premature infants on CPAP^(7)^. The major radiological differential diagnoses of PIE include cystic adenomatoid malformation, congenital lobar emphysema, congenital diaphragmatic hernia, postinfectious pneumatocele, and bronchopulmonary dysplasia^(3,5,8)^. In the present case, due to the rapid evolution of the clinical picture and the fact that the neonate had a previous radiological diagnosis of RDS, together with the fact that CPAP was used, other diagnostic hypotheses were excluded and the final diagnosis was PIE.

In conclusion, a diagnosis of PIE should always be considered when a chest X-ray of a neonate in the first hours of life shows bullous lesions not seen previously. PIE is most likely to occur in neonates submitted to any type of ventilatory support, especially in cases of extreme prematurity, in which there is a high incidence of barotrauma and severe outcomes.
